# Visit-to-visit fasting blood glucose variability and lifetime risk of cardiovascular disease: a prospective study

**DOI:** 10.1186/s12933-021-01397-1

**Published:** 2021-10-16

**Authors:** Jianing Bi, Lulu Song, Lulin Wang, Mingyang Wu, Shouhua Chen, Youjie Wang, Shouling Wu, Yaohua Tian

**Affiliations:** 1 Department of Maternal and Child Health, School of Public Health, Tongji Medical College, Huazhong University of Science and Technology, No.13 Hangkong Road, Wuhan, 430030 China; 2 Ministry of Education Key Laboratory of Environment and Health, and State Key Laboratory of Environmental Health (Incubating), School of Public Health, Tongji Medical College, Huazhong University of Science and Technology, Wuhan, China; 3 Department of Cardiology, Kailuan Hospital, North China University of Science and Technology, Tangshan City, China

**Keywords:** Fasting blood glucose variability, Cardiovascular disease, Lifetime risk

## Abstract

**Aims:**

Previous studies suggested an adverse association between higher fasting blood glucose (FBG) variability and cardiovascular disease (CVD). Lifetime risk provides an absolute risk assessment during the remainder of an individual’s life. However, the association between FBG variability and the lifetime risk of CVD is uncertain.

**Objective:**

We aimed to investigate the effect of the visit-to-visit FBG variability on the lifetime risk of CVD.

**Methods:**

This study included participants from the Kailuan Study who did not have CVD at index ages 35, 45, and 55 years. The FBG variability was defined as the coefficient of variation (CV) of three FBG values that were measured during the examination periods of 2006–2007, 2008–2009, and 2010–2011. We used a modified Kaplan-Merrier method to estimate lifetime risk of CVD according to tertiles of FBG variability.

**Results:**

At index age 35 years, the study sample comprised 46,018 participants. During a median follow-up of 7.0 years, 1889 participants developed CVD events. For index age 35 years, participants with high FBG variability had higher lifetime risk of CVD (32.5%; 95% confidence interval [CI]: 28.9–36.1%), compared with intermediate (28.3%; 95% CI: 25.5 –31.1%) and low (26.3%; 95% CI: 23.0–29.5%) FBG variability. We found that higher FBG variability was associated with increased lifetime risk of CVD in men but not women. Similar patterns were observed at index ages 45 and 55 years.

**Conclusions:**

Higher FBG variability was associated with increased lifetime risk of CVD at each index age. Focusing on the FBG variability may provide an insight to the clinical utility for reducing the lifetime risk of CVD.

**Supplementary Information:**

The online version contains supplementary material available at 10.1186/s12933-021-01397-1.

## Introduction

Cardiovascular disease (CVD) is the leading cause of death worldwide, and imposes a substantial burden on health care and the social economy [1]. Identifying and managing the risk factors of CVD are necessary for achieving primary prevention of CVD. Dysregulation of glycometabolism is a risk factor for CVD [2–4]. Traditional evaluation of dysglycaemia relies primarily on fasting blood glucose (FBG). FBG values fluctuate continuously over time. Therefore, FBG alone may not be suitable for evaluating dysglycaemia and explaining its association with CVD. FBG variability, an emerging marker of glycemic control, is a measurement that determines the fluctuations in glucose over a given time interval [5, 6]. As an integral component of glucose homoeostasis, measuring FBG variability may be an essential addition to the use of FBG in clinical practice, and may contribute to identifying those at high risk of CVD [7]. A growing body of studies suggest that visit-to-visit FBG variability is an independent predictor of CVD events in patients with diabetes or with acute coronary syndrome [8–11], while evidence on the association between visit-to-visit FBG variability and CVD in the general population is limited [12–14]. Moreover, previous studies primarily estimated the short-term relative risk of CVD, which may not provide a comprehensive assessment for the overall burden of FBG variability on CVD [15].

Lifetime risk represents a quantification of the absolute cumulative risk of developing a disease during an individual’s remaining lifespan. Estimation of the lifetime risk enables a long-term perspective for diseases and may be more easily understood by the lay public than relative risk [16–18]. To date, only one recent study evaluated the association between FBG and lifetime risk of heart failure [19]. However, no studies are available that evaluate the association between visit-to-visit FBG variability and the lifetime risk of CVD.

We thus sought to estimate the effect of visit-to-visit FBG variability on the lifetime risk of CVD. Understanding how changes in lifetime risk of CVD are associated with FBG variability may inform strategies for primary prevention of CVD, such as controlling fluctuation in FBG.

## Methods

### Study population

We used data from the Kailuan Study, which is an ongoing prospective cohort study conducted in Tangshan City, China. The detailed study design and procedures can be found elsewhere [20]. Briefly, from June 2006 to October 2007, 101,510 participants aged 18–98 years were recruited from 11 hospitals affiliated with the Kailuan Group. All participants underwent questionnaire interviews, health examinations, and laboratory assessments at enrollment and were followed up biennially since 2006.

**Fig. 1:**
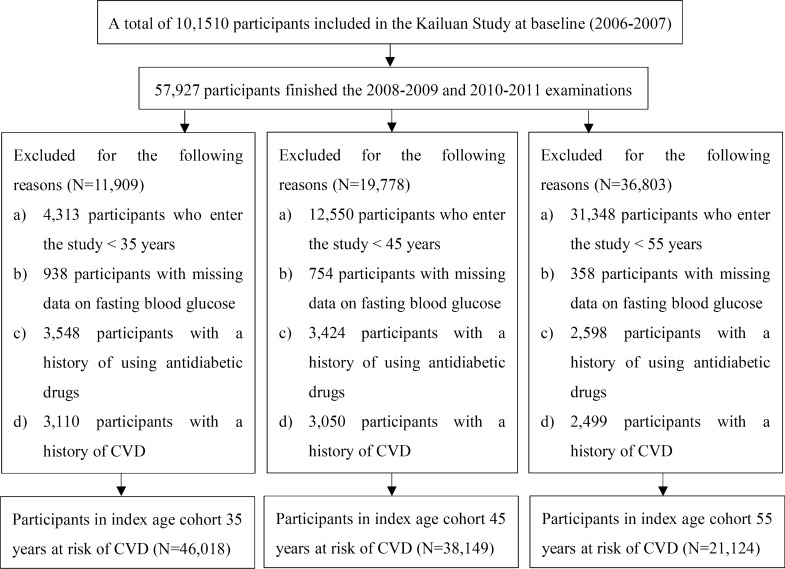
Flowchart of the study population

The study baseline was set at the date of the third examination between 2010 and 2011. A total of 57,927 participants completed the two biennial follow-up health examinations between 2008–2009 and 2010–2011. On the basis of this sample, we extracted three study samples at index ages 35, 45, and 55 years. The lifetime risk of CVD was estimated from the index age to 95 years of age. Individuals who had reached the age of 35 years when entering the study were included in the group of index age 35 group. Definitions of the group of index ages 45 and 55 years group were similar to that for the group of index age 35 years group. At index age 35 years, we excluded the participants according to the following criteria: a) attended the study at age < 35 years; (b) lack of data on FBG measurements; (c) use of antidiabetic drugs; and (d) had history of CVD before the 2010–2011. The same procedures were used to select participants for study samples at index ages 45 and 55 years. Ultimately, there were 46,018, 38,149, and 21,124 participants at index ages 35, 45 and 55 years, respectively (Fig. 1).

The study protocol was approved by the Ethics Committees of Kailuan General Hospital. Written informed consent was obtained from all the participants.

### Assessments of variables

Information on the demographic variables including age, sex, education level, smoking status, alcohol drinking status, and use of antidiabetic medications was collected from standard questionnaires. Body weight and height were measured by trained nurses according to the standard protocols. Body mass index (BMI) was calculated as weight in kilograms divided by height in meters squared. Blood samples for the measurement of FBG were drawn after an overnight fast. All measurements in blood were performed using an automatic analyzer following standard operating procedures and were subjected to regular quality control.

### Calculation of visit-to-visit FBG variability

FBG values were measured during the examination periods of from 2006 to 2007, 2008 to 2009, and 2010 to 2011. The specific time interval for FBG assessment was 2.03 ± 0.23 years. We used a common indicator of variability measurement, the coefficient of variation (CV, standard deviation [SD]/mean), to define the FBG variability. Furthermore, SD and average real variability (ARV) for FBG were also included as supplementary measures to increase the robustness of the analysis, and were calculated according to the following equations:

${\varvec{SD}} = \sqrt {\frac{{\mathop \sum \nolimits_{{{\varvec{i}} = 1}}^{{\varvec{n}}} ({\varvec{x}}_{{\varvec{i}}} - \overline{\user2{x}})^{2} }}{{{\mathbf{n}} - 1}}}$, where ${x}_{i}$ is the *i*^*th*^ point value of FBG, $\overline{x }$ is the mean of the FBG value, and n is the number of measurements for FBG.

${\varvec{ARV}} = \user2{ }\frac{1}{{{\mathbf{N}} - 1}}{{\varvec{\Sigma}}}_{{{\mathbf{k}} = 1}}^{{{\mathbf{n}} - 1}} \left| {{\mathbf{C}}_{{{\mathbf{k}} + 1}} - {\mathbf{C}}_{{\mathbf{k}}} } \right|$, where C is the value of FBG, k ranges from 1 to n−1, and n is the number of measurements for FBG [21].

Each parameter was categorized into tertiles based on the distributions in the overall sample specific to each index age. Tertile 1 was considered low FBG variability, tertile 2 was considered intermediate FBG variability, and tertile 3 was considered high FBG variability. Ranges of the categories for FBG variability were defined as follows: low FBG variability (< 6.19% for index age 35 years; < 6.26% for index age 45 years; and < 6.57% for index age 55 years); intermediate FBG variability (6.19–10.86% for index age 35 years; 6.26–11.03% for index age 45 years; and 6.57–11.40% for index age 55 years); and high FBG variability (> 10.86% for index age 35 years; > 11.03% for index age 45 years; and > 11.40% for index age 55 years).

### Outcome ascertainment

The main outcomes were incident CVD events, including myocardial infarction and stroke. Assessment of incident CVD events has been described in detail [22, 23]. Briefly, we determined CVD events from the Municipal Social Insurance Institution that covered all study participants, the discharge registers of all 11 Kailuan hospitals, death certificates, and questionnaire surveys (biennially since 2006). Incident CVD events were identified by following the *International Classification of Diseases, Tenth Revision*. To further identify potential CVD events, a panel of three experienced physicians reviewed the medical records and adjudicated the cases annually. Information on death was collected from death certificates from state vital statistics offices.

Participants in the three study samples (at index ages 35, 45, and 55 years) were followed from the baseline (2010–2011) until the occurrence of an initial CVD event or death, attainment of 95 years of age, or the end of follow up (December 31, 2017), whichever occurred first.

### Statistical analysis

Lifetime risks for initial incident CVD were estimated from index ages 35, 45, and 55 years until age 95 years. Given that the standard Kaplan–Meier method does not take into account the competing risk of death, we used a modified Kaplan–Meier method (the Practical Incidence Estimators macro) with age as the time-scale, to adjust for the competing risk of death for estimating the lifetime risk of CVD [24]. Briefly, this method accounts for the competing risks of non-cardiovascular death by treating non-cardiovascular death as a separate event rather than a withdrawal, which avoids overestimation of the lifetime risk. We estimated the lifetime risk and 95% confidence intervals (CIs) according to the categories of FBG variability (high, intermediate, and low FBG variability) at each index age, with the low FBG variability as the reference. Furthermore, we conducted an analysis stratified by sex to investigate associations between FBG variability and the lifetime risk of CVD in men and women.

All statistical analyses were conducted using SAS (version 9.4; SAS Institute Inc.). A two-tailed *P* < 0.05 was considered statistically significant.

## Results

Characteristics of the three study samples stratified by sex are shown in Table 1. Among the participants, 35,062 (76.2%), 29,316 (76.8%), and 16,570 (78.4%) were men at index ages 35, 45, and 55 years, respectively. The mean BMI in each index age group ranged from 25.0–25.2 kg/m^2^ in men, while the corresponding values in women were 24.6–25.3 kg/m^2^ for women. Compared with men, women have lower mean entry age and mean FBG variability (SD, ARV, and CV) at each index age. The median follow-up time and number of participants with first developed CVD were 7.0 years (interquartile range: 6.7–7.3) years and 1889, respectively, for index age 35 years. Corresponding values were 7.0 (6.6–7.3) years and 1790 for index age 45 years, and 6.9 (6.5–7.1) years and 1221 for index age 55 years. We compared baseline characteristics of participants who were included in and excluded from our analysis (Additional file 1: Tables S1). For most of these parameters, the differences were very small, albeit statistically significant.

**Table 1:** Characteristics of participants among men and women in three study samples

Variable	35 years		45 years		55 years	
	Men	Women	Men	Women	Men	Women
No. of participants (%)	35,062 (76.2%)	10,956 (23.8%)	29,316 (76.8%)	8833 (23.2%)	16,570 (78.4%)	4554 (21.6%)
Entry age (years)	54.8 (10.6)	52.9 (9.8)	57.6 (9.1)	56.0 (8.2)	63.6 (7.4)	62.2 (6.5)
BMI (kg/m^2^)	25.2 (3.3)	24.6 (3.6)	25.1 (3.3)	24.9 (3.6)	25.0 (3.3)	25.3 (3.6)
High school or above, n (%)	6505 (18.6)	3167 (28.9)	4753 (16.2)	2160 (24.5)	2578 (15.6)	977 (21.5)
Current smoker, n (%)	15,254 (43.5)	156 (1.4)	12,400 (42.3)	138 (1.6)	6025 (36.3)	81 (1.8)
Current alcohol drinker, n (%)	16,894 (48.2)	767 (7.0)	13,491 (46.0)	534 (6.0)	6836 (41.3)	218 (4.8)
Physical activity ≥ 3 times/week, n (%)	4839 (13.8)	1399 (12.8)	4433 (15.1)	1311 (14.8)	3561 (21.5)	991 (21.8)
Variation of FBG						
CV, %	10.13 (7.84)	9.25 (7.39)	10.23 (8.00)	9.53 (7.60)	10.32 (7.92)	10.30 (8.27)
SD (mmol/L)	0.59 (0.92)	0.51 (0.86)	0.60 (0.98)	0.53 (0.91)	0.60 (1.07)	0.59 (1.14)
ARV (mmol/L)	0.72 (1.19)	0.62 (1.17)	0.73 (1.28)	0.65 (1.20)	0.73 (1.37)	0.72 (1.57)

Data was presented as mean (SD); *BMI* body mass index, *FBG* Fasting blood glucose, *CV* Coefficient of variation, SD Standard deviation, *ARV* Average real variability

**Fig. 2:**
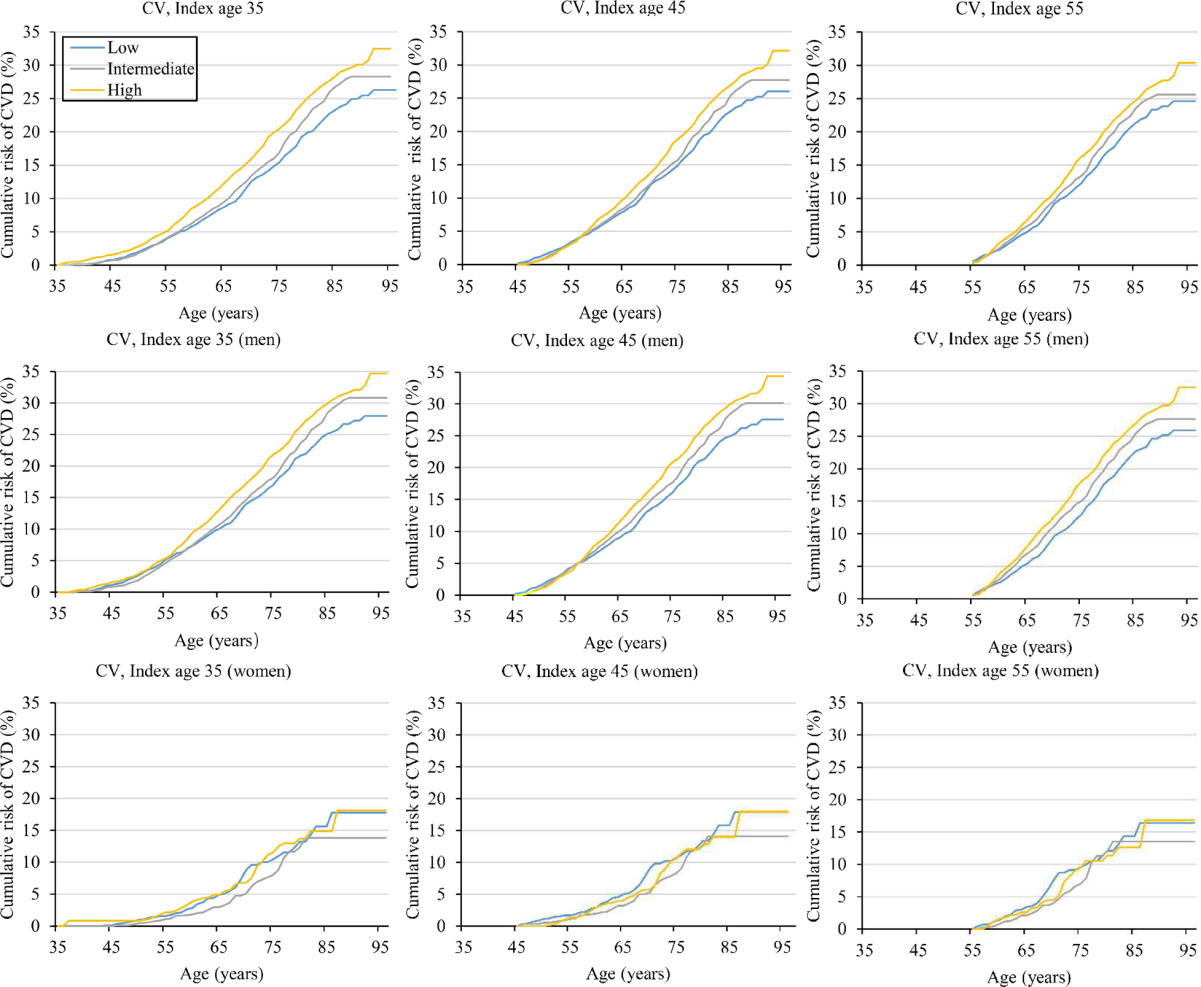
The lifetime risk of CVD adjusted for the competing risk of death for men and women at ages 35, 45 and 55 according to the categories of fasting blood glucose variability (CV). *CVD* Cardiovascular diseases, *CV* Coefficient of variation

Table 2 and Fig. 2 showed the lifetime risk of CVD (up to age 95 years) at index ages 35, 45, and 55 years according to the categories of FBG variability, after adjustment for competing risk of death. In each index age, individuals with high FBG variability (CV) had higher lifetime risk of CVD compared with those with intermediate or low FBG variability in each index age. For example, at index age 35 years, the lifetime risks of CVD were 26.3% (95% CI: 23.0%–29.5%), 28.3% (95% CI: 25.5%–31.1%) and 32.5% (95% CI: 28.9%–36.1%) for individuals with low, intermediate, and high FBG variability, respectively. We also calculated the lifetime risk of CVD in men and women according to the categories of FBG variability (Table 2 and Fig. 2). Among men at index age 35 years, those with high FBG variability had higher lifetime risk of CVD (34.7%; 95% CI: 30.9–38.5%) than those with intermediate (30.8%; 95% CI: 27.9–33.8%) or low (28.0%; 95% CI: 24.5–31.4%) FBG variability. Among women, no significant association was found between the FBG variability and the lifetime risk of CVD. Patterns were similar at index ages 45 and 55 years (Table 2 and Fig. 2).

**Table 2:** The lifetime risk of CVD up to age 95 adjusted for the competing risk of death for men and women at age 35, 45 and 55 according to individual fasting blood glucose variability (CV)

FBG variability category (CV, %)	Total			Men			Women		
	No. of CVD case/total	Lifetime risk% (95%CI)	*P*-value	No. of CVD case/total	Lifetime risk% (95%CI)	*P*-value	No. of CVD case/total	Lifetime risk% (95%CI)	*P*-value
35 years									
Low (< 6.19)	528/15339	26.3 (23.0, 29.5)	Ref	455/11262	28.0 (24.5, 31.4)	Ref	73/4077	17.8 (11.3, 24.2)	Ref
Intermediate (6.19–10.86)	603/15340	28.3 (25.5, 31.1)	0.357	547/11676	30.8 (27.9, 33.8)	0.214	56/3664	13.8 (9.6, 18.1)	0.316
High (> 10.86)	758/15339	32.5 (28.9, 36.1)	0.013	688/12124	34.7 (30.9, 38.5)	0.010	70/3215	18.1 (10.6, 25.7)	0.942
45 years									
Low (< 6.26)	502/12713	26.0 (22.8, 29.3)	Ref	430/9519	27.5 (24.1, 31.0)	Ref	72/3194	17.9 (11.5, 24.2)	Ref
Intermediate (6.26%-11.03%)	584/12720	27.7 (25.0, 30.5)	0.443	528/9755	30.1 (27.2, 33.1)	0.269	56/2965	14.1 (9.9, 18.4)	0.335
High (> 11.03%)	704/12716	32.1 (28.3, 35.9)	0.018	638/10042	34.4 (30.5, 38.4)	0.011	66/2674	18.0 (9.4, 26.5)	0.990
55 years									
Low (< 6.57%)	345/7041	24.6 (21.3, 28.0)	Ref	294/5533	25.9 (22.3, 29.5)	Ref	51/1508	16.4 (10.2, 22.5)	Ref
Intermediate (6.57–11.40)	408/7042	25.6 (22.8, 28.4)	0.655	364/5505	27.6 (24.5, 30.7)	0.468	44/1537	13.5 (9.1, 18.0)	0.464
High (> 11.40)	468/7041	30.4 (26.3, 34.5)	0.032	423/5532	32.5 (28.2, 36.8)	0.020	45/1509	16.8 (7.8, 25.8)	0.937

*CVD* Cardiovascular diseases, *FBG* Fasting blood glucose, *CV* Coefficient of variation

Lifetime risk estimates represent the percentage of cohort participants who would experience a total CVD event from the index age to the end of follow-up if the last participant in the cohort were to die at the last age of follow-up (95 years)

To further evaluate the robustness of the analysis, SD and ARV of FBG were used to replace CV in all the models, and the analyses yielded similar findings (Additional file 1: Tables S2, S3 and Figures S1, S2).

## Discussion

In the present study, we estimated the lifetime risk of CVD adjusted for the competing risk of death in total, male, and women participants at index ages 35, 45, and 55 years according to categories of FBG variability. We found that across different index ages high FBG variability was associated with increased lifetime risk of CVD compared with low FBG variability, and this association was not significant in women.

Previous studies have suggested an adverse effect of high FBG variability on CVD in high risk populations, such as patients with diabetes or acute coronary syndrome [8–10]. However, whether high FBG variability can increase the risk of CVD in the general population remains uncertain. To date, only three studies have explored the association between FBG variability and CVD in the general population, and have yielded inconsistent results [12–14]. Two cohort studies, conducted in Korea and China, found that high FBG variability was associated with an increased risk of CVD [12, 13]. In contrast, another cohort study conducted in the United States reported no excess risk of CVD in individuals with high FBG variability [14]. Furthermore, all previous studies were limited by relative risk of CVD, which could not provide a long-term and much more accessible descriptions of the effect of the FBG variability on CVD [15]. Our findings expand on the prior analyses that quantified the absolute risk of FBG variability on CVD during the remaining life of an individual. We found that individuals with high FBG variability had greater lifetime risk of CVD than those with intermediate or low FBG variability at each index age.

Although the potential mechanisms underlying the association between FBG variability and lifetime risk of CVD are unclear, our findings are plausible. Experimental studies in vivo and in vitro have found that, compared with stable glucose levels, glucose fluctuation was associated with significantly higher levels of oxidative stress markers and inflammatory cytokines and was accompanied by impaired endothelial function [25–27]. These pathophysiological changes are the major triggers for CVD [28]. Moreover, a clinical study found that high glycemic variability was associated with an increased risk of thrombosis [29], which may directly promote the development of CVD [30, 31].

We further found that the positive association between FBG variability and the lifetime risk of CVD was insignificant in women. A possible explanation for the sex-specific observations may be the higher estrogen level in women [32–34]. Studies have suggested that higher estrogen levels can up-regulate the expression of antioxidant genes [35, 36] and inhibit the expression of pro-inflammatory genes [37]. Furthermore, estrogen has known anti-apoptotic properties [38, 39]. For example, 17β-estradiol, the most active metabolite of estrogen, can prevent both endothelial cell and endothelial progenitor cell apoptosis [40, 41]. Therefore, because of estrogen protection, women may be more adaptable to FBG variability than men, which may reduce the risk of CVD caused by FBG variability.

The findings from our study may be useful for formulating the glycaemia management strategies for the primary prevention of CVD. The present data showed that high FBG variability can increase the risk of CVD, which suggests it is important for individuals to maintain stable FBG levels in daily life. Moreover, newly updated clinical practice guidelines recommended that the primary purpose of assessment of CVD risk is to provide the basis for a discussion of risk with patients [42]. Our estimates for the effect of FBG variability on the lifetime risk of CVD can be applied to extend the existing counselling of patients and for decision making, which are currently based on the relative risk of CVD alone.

Strengths of the present study include its prospective design and large sample size, and that the association between visit-to-visit FBG variability and lifetime risk of CVD was estimated for the first time. However, several limitations should also be noted. First, the observational study design limited the ability to establish causal pathways. Second, we did not measure other parameters that also reflect blood glucose variability, such as glycated hemoglobin and continuous glucose monitoring. Third, although we excluded patients taking antidiabetic drugs, we could not fully eliminate some unmeasured confounding factors, which may blunt the FBG variability, such as diet management. Fourth, FBG values were only measured for three times and the median follow-up time was approximately 7 years, which is relatively short. Finally, all the participants in our study were Chinese, which may limit the generalizability of the results of our investigation.

## Conclusions

In conclusion, our findings emphasized the adverse effect of high FBG variability on the lifetime risk of CVD, particularly in men. These findings may contribute to refining the preventative strategies for CVD and motivate individuals to effectively maintain stable FBG.

## Supplementary Information

**Additional file 1. Table S1. **Characteristics of the participants who were included in and excluded from the analysis. **Table S2. **The lifetime risk of CVD up to age 95 adjusted for the competing risk of death for men and women at age 35, 45 and 55 according to the categories of fasting blood glucose variability (ARV). **Table S3. **The lifetime risk of CVD up to age 95 adjusted for the competing risk of death for men and women at ages 35, 45 and 55 according to the categories of fasting blood glucose variability (SD). **Figure S1. **The lifetime risk of CVD adjusted for the competing risk of death for men and women at age 35, 45 and 55 according to individual fasting blood glucose variability (ARV). **Figure S2. **The lifetime risk of CVD adjusted for the competing risk of death for men and women at age 35, 45 and 55 according to individual fasting blood glucose variability (SD).

## Data Availability

The data that support the findings of this study are available from the corresponding author upon reasonable request.

## Abbreviations

FBG: Fasting blood glucose
CVD: Cardiovascular disease
CI: Confidence interval
BMI: Body mass index
CV: Coefficient of variation
SD: Standard deviation
ARV: Average real variability
