# Supplementary material for: Visit-to-visit fasting blood glucose variability and lifetime risk of cardiovascular disease: a prospective study
Source: Cardiovasc Diabetol. 2021 Oct 16;20:207. doi: 10.1186/s12933-021-01397-1 (PMC8520235; doi:10.1186/s12933-021-01397-1)
Supplement: Supplementary file 1 — Additional file 1. Table S1. Characteristics of the participants who were included in and excluded from the analysis. Table S2. The lifetime risk of CVD up to age 95 adjusted for the competing risk of death for men and women at age 35, 45 and 55 according to the categories of fasting blood glucose variability (ARV). Table S3. The lifetime risk of CVD up to age 95 adjusted for the competing risk of death for men and women at ages 35, 45 and 55 according to the categories of fasting blood glucose variability (SD). Figure S1. The lifetime risk of CVD adjusted for the competing risk of death for men and women at age 35, 45 and 55 according to individual fasting blood glucose variability (ARV). Figure S2. The lifetime risk of CVD adjusted for the competing risk of death for men and women at age 35, 45 and 55 according to individual fasting blood glucose variability (SD). [file 12933_2021_1397_MOESM1_ESM.docx]

**Supplemental materials**

Title：Visit-to-visit fasting blood glucose variability and lifetime risk of cardiovascular disease: A prospective study.

Table and figure legends:

Table S1. Characteristics of the participants who were included in and excluded from the analysis.

Table S2. The lifetime risk of CVD up to age 95 adjusted for the competing risk of death for men and women at age 35, 45 and 55 according to the categories of fasting blood glucose variability (ARV).

Table S3. The lifetime risk of CVD up to age 95 adjusted for the competing risk of death for men and women at ages 35, 45 and 55 according to the categories of fasting blood glucose variability (SD).

Figure S1. The lifetime risk of CVD adjusted for the competing risk of death for men and women at age 35, 45 and 55 according to individual fasting blood glucose variability (ARV).

Figure S2. The lifetime risk of CVD adjusted for the competing risk of death for men and women at age 35, 45 and 55 according to individual fasting blood glucose variability (SD).

Table S1. Characteristics of the participants who were included in and excluded from the analysis.

| Characteristics | Included (n=57,927) | Excluded (n=43,583) | *P-*value |
| --- | --- | --- | --- |
| Age (years) | 49.4 (12.0) | 55.2 (12.9) | <0.01 |
| Sex, men | 44,332 (76.5%) | 36,778 (84.3%) | <0.01 |
| High school or above, n (%) | 13952 (24.1) | 6510 (14.9) | <0.01 |
| Current smoker, n (%) | 19495 (33.7) | 14300 (32.8) | 0.069 |
| Current alcohol drinker, n (%) | 22348 (38.6) | 14304 (32.8) | <0.01 |
| Physical activity≥ 3 times/week, n (%) | 8186 (14.1) | 7095 (16.3) | <0.01 |
| BMI (BMI (kg/m2) | 25.1 (3.5) | 25.0 (3.5) | <0.01 |
| FBG (mmol/L) | 5.4 (1.6) | 5.6 (1.8) | <0.01 |

Note: Data was presented as mean (SD); BMI: Body mass index; FBG: Fasting blood glucose.

Table S2. The lifetime risk of CVD up to age 95 adjusted for the competing risk of death for men and women at age 35, 45 and 55 according to the categories of fasting blood glucose variability (ARV).

| FBG variability category (ARV, mmol/L) | Total | | |  | Men | | | | |  | Women | | | |
| --- | --- | --- | --- | --- | --- | --- | --- | --- | --- | --- | --- | --- | --- | --- |
|  | No. of CVD case/total | Lifetime risk  % (95%CI) | *P*-value |  | No. of CVD case/total | | Lifetime risk  % (95%CI) | *P*-value | |  | No. of CVD case/total | | Lifetime risk  % (95%CI) | *P*-value |
| 35 years |  |  |  |  |  |  | | |  |  |  |  | |  |
| Low (< 0.38) | 514/15387 | 26.3 (23.0, 29.5) | Ref |  | 442/11187 | 28.3 (24.8, 31.7) | | | Ref |  | 75/4232 | 17.1 (10.9, 23.4) | | Ref |
| Intermediate (0.38－0.70) | 624/15308 | 28.6 (25.9, 31.2) | 0.275 |  | 558/11622 | 31.0 (28.2, 33.8) | | | 0.231 |  | 53/3677 | 13.5 (9.2, 17.9) | | 0.350 |
| High (> 0.70) | 751/15323 | 32.1 (28.4, 35.7) | 0.019 |  | 690/12253 | 34.1 (30.4, 37.9) | | | 0.025 |  | 71/3047 | 19.1 (11.6, 26.7) | | 0.692 |
| 45 years |  |  |  |  |  |  | | |  |  |  |  | |  |
| Low (< 0.39) | 493/12754 | 26.1 (22.8, 29.3) | Ref |  | 422/9458 | 28.0 (24.5, 31.6) | | | Ref |  | 71/3296 | 16.1 (10.5, 21.7) | | Ref |
| Intermediate (0.39－0.70) | 593/12600 | 28.0 (25.3, 30.7) | 0.372 |  | 530/9667 | 30.2 (27.3, 33.0) | | | 0.35 |  | 63/2933 | 14.7 (10.0, 19.4) | | 0.716 |
| High (> 0.70) | 704/12795 | 31.8 (27.9, 35.6) | 0.027 |  | 644/10191 | 33.9 (29.9, 37.9) | | | 0.032 |  | 60/2604 | 19.9 (10.7, 29.1) | | 0.488 |
| 55 years |  |  |  |  |  |  | | |  |  |  |  | |  |
| Low (< 0.40) | 322/7066 | 23.7 (20.4, 27.0) | Ref |  | 283/5510 | 25.1 (21.5, 28.7) | | | Ref |  | 49/1556 | 15.0 (9.4, 20.6) | | Ref |
| Intermediate (0.40－0.72) | 426/7018 | 27.2 (24.4, 30.1) | 0.112 |  | 376/5511 | 29.2 (26.1, 32.4) | | | 0.087 |  | 50/1507 | 14.7 (9.6, 19.8) | | 0.936 |
| High (>0.72%) | 463/7040 | 30.1 (25.6, 34.6) | 0.024 |  | 422/5549 | 32.1 (27.5, 36.8) | | | 0.018 |  | 41/1491 | 17.9 (8.5, 27.3) | | 0.610 |

Note: CVD, Cardiovascular diseases; FBG, Fasting blood glucose; ARV, Average real variability.

Lifetime risk estimates represent the percentage of cohort participants who would experience a total CVD event from the index age to the end of follow-up if the last participant in the cohort were to die at the last age of follow-up (95 years).

Table S3. The lifetime risk of CVD up to age 95 adjusted for the competing risk of death for men and women at ages 35, 45 and 55 according to the categories of fasting blood glucose variability (SD).

| FBG variability category (SD, mmol/L) | Total | | |  | Men | | | | |  | Women | | | |
| --- | --- | --- | --- | --- | --- | --- | --- | --- | --- | --- | --- | --- | --- | --- |
|  | No. of CVD case/total | Lifetime risk  % (95%CI) | *P*-value |  | No. of CVD case/total | | Lifetime risk  % (95%CI) | *P*-value | |  | No. of CVD case/total | | Lifetime risk  % (95%CI) | *P*-value |
| 35 years |  |  |  |  |  |  | | |  |  |  |  | |  |
| Low (< 0.32) | 517/15321 | 26.3 (23.0, 29.6) | Ref |  | 442/11089 | 28.1 (24.7, 31.6) | | | Ref |  | 75/4232 | 17.1 (10.9, 23.4) | | Ref |
| Intermediate (0.32－0.57) | 590/15341 | 27.3 (24.6, 30.1) | 0.630 |  | 537/11664 | 29.8 (26.9, 32.7) | | | 0.478 |  | 53/3677 | 13.5 (9.2, 17.9) | | 0.350 |
| High (> 0.57) | 782/15356 | 33.3 (29.7, 36.8) | 0.005 |  | 711/12309 | 35.2 (31.6, 38.8) | | | 0.006 |  | 71/3047 | 19.1 (11.6, 26.7) | | 0.692 |
| 45 years |  |  |  |  |  |  | | |  |  |  |  | |  |
| Low (< 0.32) | 499/12718 | 25.9 (22.7, 29.1) | Ref |  | 421/9422 | 27.4 (23.9, 30.9) | | | Ref |  | 78/3296 | 17.9 (11.8, 24.0) | | Ref |
| Intermediate (0.32－0.58) | 574/12716 | 27.3 (24.6, 30.1) | 0.513 |  | 526/9724 | 29.8 (26.9, 32.8) | | | 0.292 |  | 48/2992 | 12.7 (8.5, 17.0) | | 0.174 |
| High (> 0.58) | 717/12715 | 32.5 (28.8, 36.3) | 0.008 |  | 649/10170 | 34.4 (30.7, 38.1) | | | 0.007 |  | 68/2545 | 18.8 (11.1, 26.6) | | 0.851 |
| 55 years |  |  |  |  |  |  | | |  |  |  |  | |  |
| Low (< 0.35) | 339/7040 | 23.9 (20.6, 27.2) | Ref |  | 285/5485 | 25.1 (21.6, 28.7) | | | Ref |  | 54/1555 | 16.1 (10.0, 22.1) | | Ref |
| Intermediate (0.35－0.60) | 400/7043 | 25.5 (22.6, 28.4) | 0.479 |  | 362/5513 | 27.6 (24.5, 30.8) | | | 0.297 |  | 38/1530 | 12.7 (8.2, 17.1) | | 0.375 |
| High (>0.60%) | 482/7041 | 31.0 (27.0, 35.0) | 0.007 |  | 434/5572 | 32.8 (28.8, 36.8) | | | 0.005 |  | 48/1469 | 17.8 (9.4, 26.2) | | 0.744 |

Note: CVD, Cardiovascular diseases; FBG, Fasting blood glucose; SD, Standard deviation.

Lifetime risk estimates represent the percentage of cohort participants who would experience a total CVD event from the index age to the end of follow-up if the last participant in the cohort were to die at the last age of follow-up (95 years).

Figure S1. The lifetime risk of CVD adjusted for the competing risk of death for men and women at age 35, 45 and 55 according to individual fasting blood glucose variability (ARV).


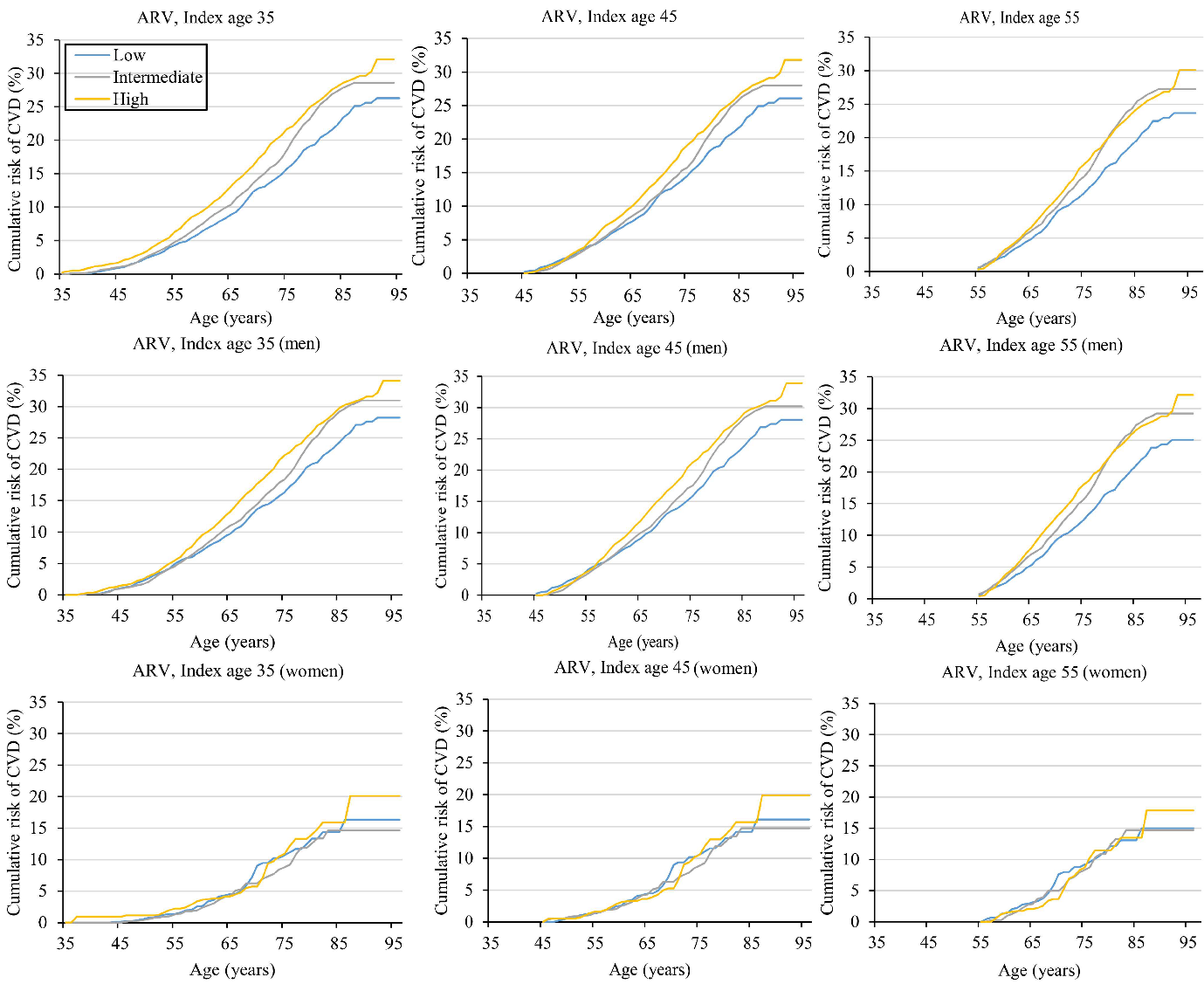


Note: CVD, Cardiovascular diseases; ARV, Average real variability.

Figure S2. The lifetime risk of CVD adjusted for the competing risk of death for men and women at age 35, 45 and 55 according to individual fasting blood glucose variability (SD).


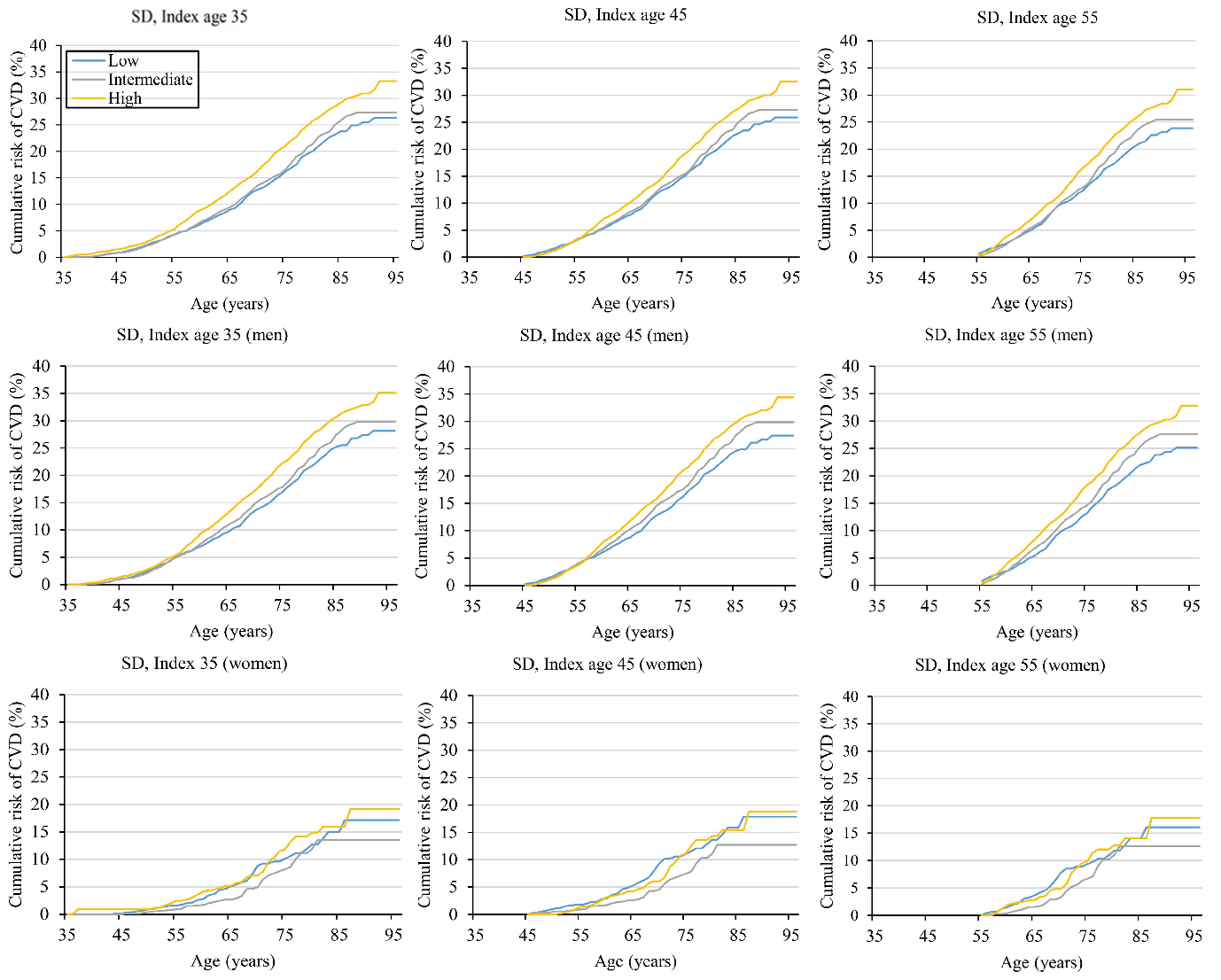


Note: CVD, Cardiovascular diseases; SD, Standard deviation.
